# Knockdown of hsa_circ_0091994 constrains gastric cancer progression by suppressing the miR-324-5p/HMGA1 axis

**DOI:** 10.18632/aging.203450

**Published:** 2021-08-24

**Authors:** Yi Xie, Zhao Liu, Hanfang Zhu

**Affiliations:** 1 Department of Gastrointestinal Surgery, Henan Provincial People's Hospital, Zhengzhou 450000, Henan, China

**Keywords:** hsa_circ_0091994 (cicrRNA_105040), gastric cancer, miR-324-5p, apoptosis, migration

## Abstract

CircRNAs have emerged as potential therapeutic targets for diseases such as gastric cancer (GC). We identified highly dysregulated circRNAs in GC tissue and further explored their potential mechanisms in the progression of GC. Hsa_circ_0091994 (cicrRNA_105040) was identified as a highly upregulated circRNA in GC tissues, whose host gene is negatively associated with the overall survival of patients. Using cell counting kit-8 and Annexin V assays, we observed that hsa_circ_0091994 knockdown inhibited the viability of AGS and HGC-27 cells by inducing apoptosis. Scratch wound healing assays showed that hsa_circ_0091994 knockdown also inhibited GC cell healing. Bioinformatics analysis and a luciferase assays revealed that hsa_circ_0091994 knockdown inhibits GC progression by suppressing miR-324-5p and HMGA1 expression. The antitumor effect of hsa_circ_0091994 knockdown was confirmed *in vivo* using a mouse xenograft model. Hsa_circ_0091994 knockdown inhibited the progression of GC by inhibiting the miR-324-5p/HMGA1 axis.

## INTRODUCTION

Gastric cancer (GC) is a prevalent malignancy with nearly 1 million new cases per year globally [1–4]. China has the highest incidence of GC, and more than 50% of cases occur in East Asia [5–7]. GC is the third leading cause of cancer mortality worldwide with over 720,000 deaths annually [7, 8]. Surgery remains the primary treatment strategy [3], but the 5-year survival rate is only 20-50% with surgery alone [9]. Even with multimodal treatment, the 5-year overall survival rate for patients with advanced GC is less than 5% [3].

Dysregulation of several genes and pathways are correlated to the pathogenesis of GC [10, 11]. Circular RNA (circRNA) is a class of endogenous RNA with covalently-closed loop structures [12]. Back-spliced exons generate circRNAs throughout the eukaryotic transcriptome [13]. During the post-transcriptional process, circRNAs can disrupt miRNAs [14]. CircRNAs stimulate GC cell proliferation, apoptosis, migration, invasion, and epithelial mesenchymal transition (EMT) [15, 16]. CircRNAs also promote the initiation, development, differentiation, metastasis, and TNM stage of GC tumors [15, 17].

Using the Gene Expression Omnibus (GEO) dataset, we compared the expression profiling of circRNAs between GC tissues and normal tissues. Differentially expressed circRNAs (DEcircRNAs) of GSE141977 were explored *in vitro* and *in vivo*.

## RESULTS

### CircRNA hsa_circ_0091994 is increased in GC tissue

**Figure 1:**
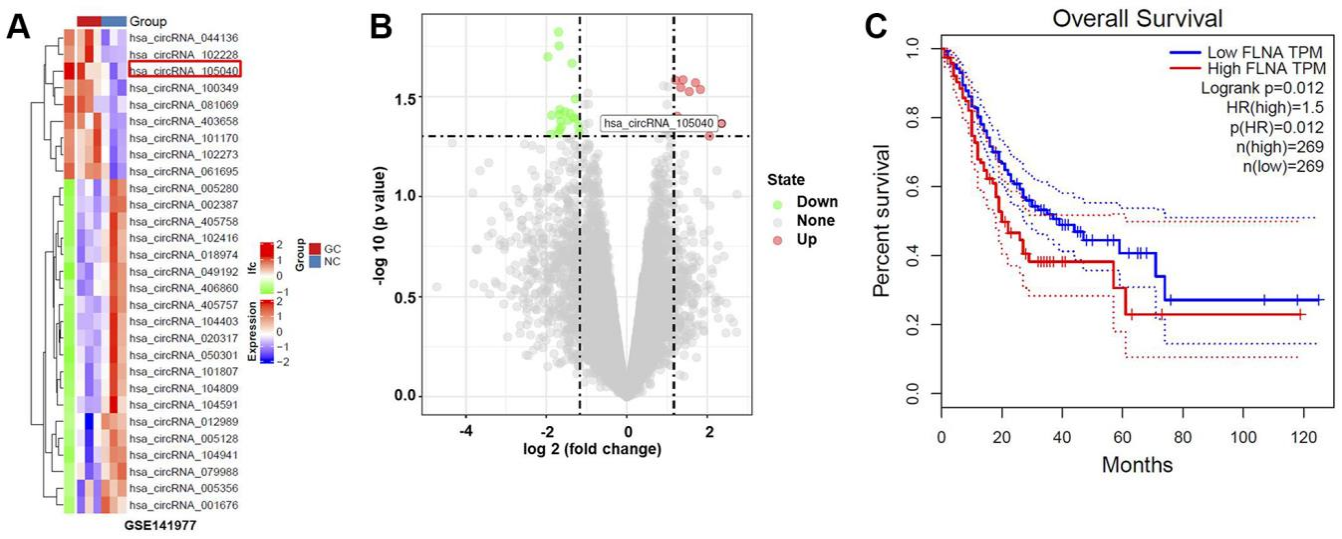
**Bioinformatics analysis to identify DEcircRNAs in GC.** (**A**) Heat map indicating DEcircRNAs in GC tissue. (**B**) Volcano plot of DEcircRNAs. Highly downregulated and upregulated genes indicated in green and red, respectively. P-value < 0.05 (-log_10_ p-value > 1.3) and |log_2_(fold change)| > 1.5 were thresholds. (**C**) Survival analysis demonstrating the relationship between the level of FLNA (the host gene of hsa_circ_0091994) and survival rate of GC patients.

We identified dysregulated circRNAs in GC tissues using the GSE141977 dataset (Figure 1A, 1B). The association between the host genes of these dysregulated circRNAs and the survival rate of patients with GC was analyzed. FLNA, the host gene of hsa_circ_0091994 (circ_105040), was negatively correlated to the survival rate of patients with GC (Figure 1C).

### Hsa_circ_0091994 knockdown repressed cell viability of AGS and HGC-27

**Figure 2:**
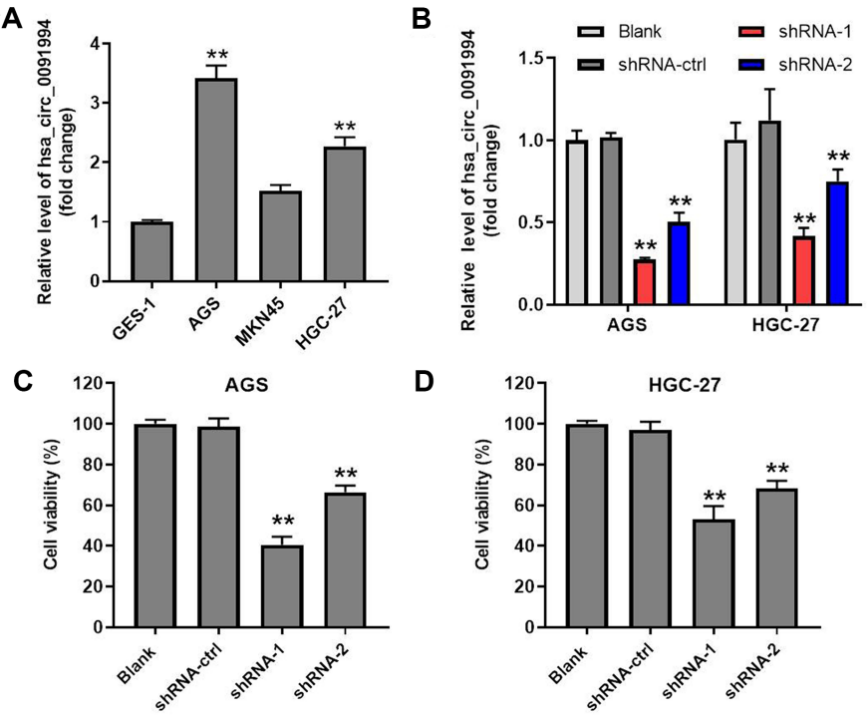
**Hsa_circ_0091994 knockdown inhibited cell viability of AGS and HGC-27.** (**A**) The relative level of hsa_circ_0091994 in human gastric epithelial cell line GES-1 and in GC cell lines including AGS, MKN45, and HGC-27. **P<0.01, compared with GES-1; n = 3. (**B**) AGS and HGC-27 cells were administrated with shRNA-ctrl, shRNA-1 or shRNA-2 of hsa_circ_0091994 for 48 hr. The efficacy of hsa_circ_0091994 knockdown was evaluated by RT-qPCR. (**C**, **D**) The effect of shRNA-1 or shRNA-2 of hsa_circ_0091994 on the viability of AGS and HGC-27 cells were determined by CCK-8 assay. **P<0.01, compared with blank; n = 3.

Levels of hsa_circ_0091994 in GES-1, GAS, MKN45, and HGC-27 cells were detected by RT-qPCR. Hsa_circ_0091994 expression was increased in three GC cancer cell lines compared with GES-1 (Figure 2A). Compared with MKN45, hsa_circ_0091994 had higher levels in AGS cells and HGC-27 cells. Therefore, AGS and HGC-27 cells were selected for exploring the role of hsa_circ_0091994 *in vitro*.

Two shRNAs (shRNA1 and shRNA2) were designed and synthesized to knockdown hsa_circ_0091994 in AGS cells and HGC-27 cells, and knockdown efficacy was confirmed with RT-qPCR (Figure 2B). Cell viability assay was performed at 48 hr after transfection, which showed that both hsa_circ_0091994 shRNAs suppressed gastric cancer cell viability (Figure 2C, 2D and Supplementary Figure 1A). However, hsa_circ_0091994 shRNA-1 exhibited a stronger inhibition of cell viability and was carried forward into the next experiments. In contrast, overexpression of hsa_circ_0091994 significantly promoted the proliferation of gastric cancer cells (Supplementary Figure 1B, 1C).

### Hsa_circ_0091994 knockdown induced apoptosis and inhibited wound healing activity in AGS and HGC-27 cells

**Figure 3:**
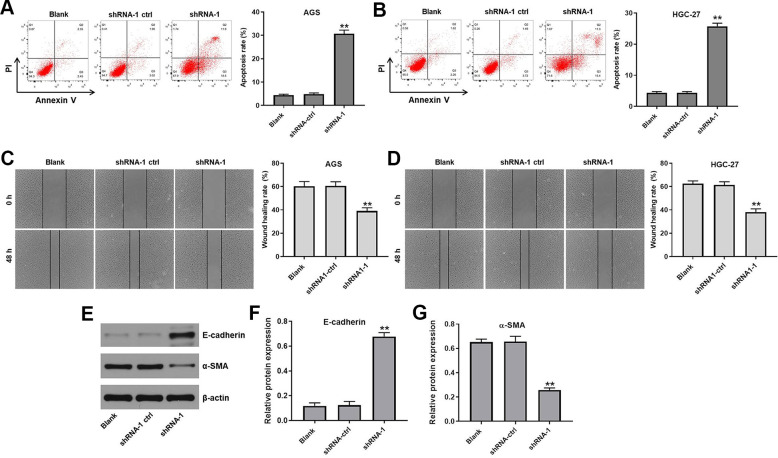
**Hsa_circ_0091994 knockdown promoted the apoptosis and suppressed the wound healing activity in AGS and HGC-27 cells.** AGS and HGC-27 cells were treated with shRNA-1 or shRNA-ctrl for 48 hr. (**A**, **B**) Cell apoptosis rate was measured in each group. (**C**, **D**) Wound healing assay was employed to evaluate the effect of hsa_circ_0091994 knockdown on wound healing activity of AGS and HGC-27 cell. (**E**) The protein expression of E-cadherin and α-SMA was examined using western blot assay. β-actin was used as a loading control. (**F**, **G**) The expressions of E-cadherin and α-SMA were quantified respectively. The**P<0.01, compared with blank; n = 3.

We used Annexin V and PI double staining to assess the effect of hsa_circ_0091994 knockdown on GC cell apoptosis. Compared with shRNA-ctrl group, shRNA-1 stimulated apoptosis in gastric cancer cells (Figure 3A, 3B and Supplementary Figure 1D). In addition, the wound healing activity of AGS and HGC-27 cells were inhibited by hsa_circ_0091994 shRNA-1 (Figure 3C, 3D). Meanwhile, the expression of EMT associated makers in AGS cells was detected with western blot. The result indicated hsa_circ_0091994 knockdown increased the level of E-cadherin and decreased the level of α-SMA (Figure 3E–3G). Taken together, hsa_circ_0091994 knockdown induced apoptosis and inhibited wound healing activity in AGS and HGC-27 cells.

### Hsa_circ_0091994 functions through regulating miR-324-5p/HMGA1 axis

**Figure 4:**
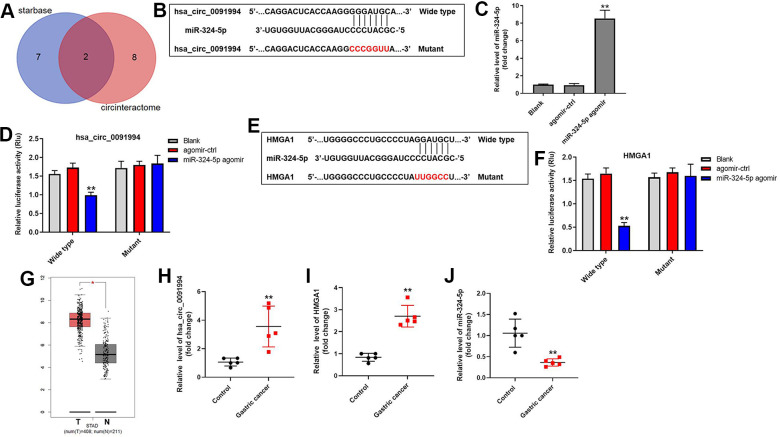
**Downstream target of hsa_circ_0091994.** (**A**) The downstream targets of hsa_circ_0091994 were predicted using Starbase and CircInteractome. (**B**) MiR-324-5p was predicted as the downstream target of hsa_circ_0091994. Sequences of wild type and mutant 3’ UTR of miR-324-5p. (**C**) MiR-324-5p agomir or the negative control (agomir-ctrl) was transfected into AGS cells for 48 hr. The efficacy of miR-324-5p agomir was detected by RT-qPCR. (**D**) Dual luciferase assay for hsa_circ_0091994 was conducted at 48 hr after transfection. (**E**) HMGA1 was predicted as the downstream target of miR-324-5p. Sequences of wild type and mutant 3’ UTR of HMGA1 were presented. (**F**) Dual luciferase assay for HMGA1 was conducted at 48 hr after transfection. (**G**) Bioinformatics analysis using GEPIA tool was performed to analyze the levels of HMGA1 in GC tissue and normal gastric tissue. (**H**–**J**). The expressions of hsa_circ_0091994, miR-324-5p and HMGA1 in 5 pairs of clinical GC and adjacent normal tissues were detected with RT-qPCR. **P<0.01, compared with blank; n = 3.

In order to investigate the underlying mechanisms by which hsa_circ_0091994 regulated GC progression, the putative targets of hsa_circ_0091994 were predicted using StarBase and CircInteractome databases. MiR-324-5p and miR-217 were predicted as potential targets of hsa_circ_0091994 (Figure 4A). Among these two miRNAs, miR-324-5p is correlated with GC progression [18], so we investigated the relationship between hsa_circ_0091994 and miR-324-5p. Wild type and mutant 3’ UTR of hsa_circ_0091994 were listed and synthesized (Figure 4B), and miR-324-5p agomir or negative control (agomir control) was transfected into the cells (Figure 4C). The relative luciferase activity of wild type hsa_circ_0091994 was decreased by miR-324-5p agomir, indicating that miR-324-5p was the target of hsa_circ_0091994 (Figure 4D). In addition, the result of RNA pull-down indicated miR-324-5p directly binding to hsa_circ_0091994 (Supplementary Figure 2A). Moreover, miR-324-5p antagomir reversed the effects of hsa_circ_0091994 knockdown on cell viability and apoptosis (Supplementary Figure 2B–2D).

We further explored the downstream target of miR-324-5p. HMGA1 was predicted as one of the putative downstream targets of miR-324-5p (Figure 4E), and HMGA1 promotes GC progression [19]. Relative luciferase activity of wild type HMGA1 was decreased by miR-324-5p agomir (Figure 4F), suggesting HMGA1 is a downstream target of miR-324-5p.

HMGA1 expression in GC tissue and adjacent normal tissue was analyzed using GEPIA tool based on the expression profile from TCGA database [20]. Bioinformatics analysis showed that HMGA1 was highly expressed in GC tumor tissue (Figure 4G). In addition, the expressions of hsa_circ_0091994 and HMGA1 in clinical gastric tumor tissues were notably upregulated, while the level of miR-324-5p was downregulated compared with that in adjacent tissues (Figure 4H–4J). All these data suggested hsa_circ_0091994 functions through regulating miR-324-5p/HMGA1 axis.

### HMGA1 OE reversed the effects of hsa_circ_0091994 knockdown on cell viability and apoptosis

**Figure 5:**
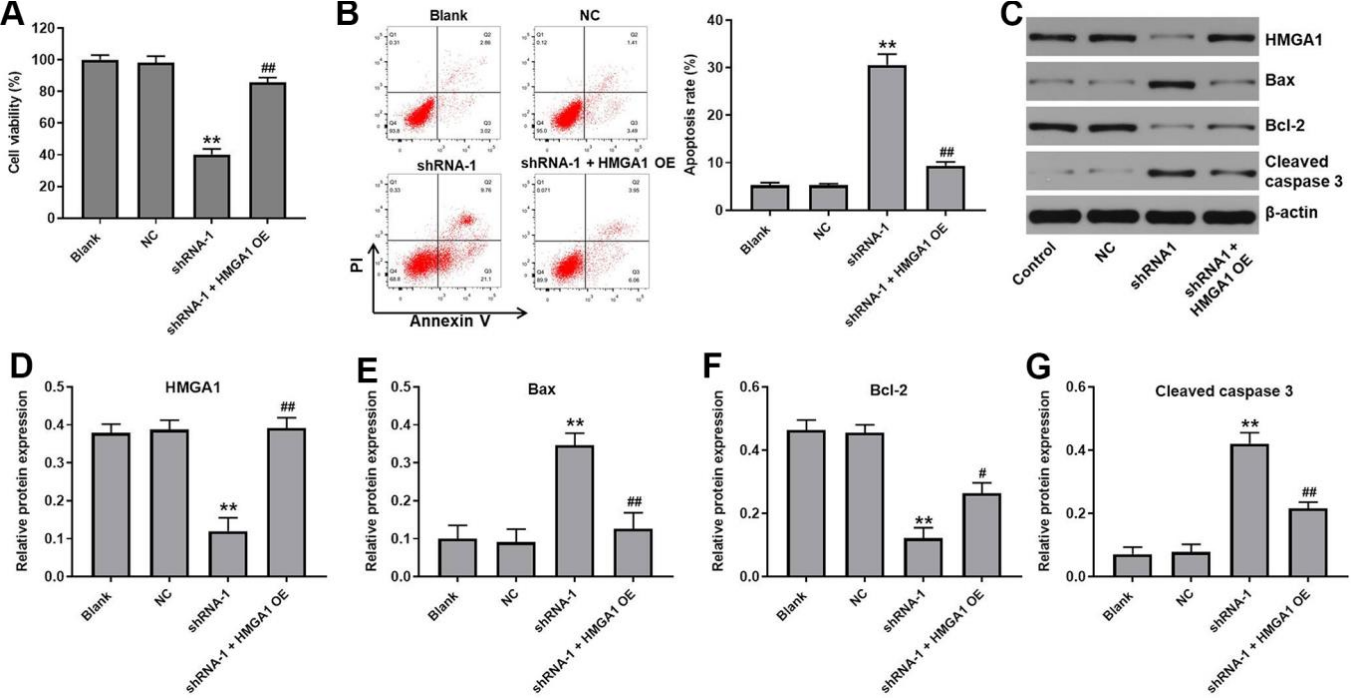
**HMGA1 OE reversed the effect of hsa_circ_0091994 knockdown on the cell viability and apoptosis.** AGS cells were administrated with shRNA-1 and HMGA1 OE for 48 h. (**A**) Cell viability was detected using CCK-8 assay. (**B**) Cell apoptosis was detected using Annexin V and PI double staining; cell apoptosis rate was quantified. (**C**) The protein expression of HMGA1, Bax, Bcl-2, cleaved caspase 3 was examined using western blot assay. β-actin was used as a loading control. (**D**–**G**) The protein expressions of HMGA1, Bax, Bcl-2, and cleaved caspase 3 were quantified respectively. **P<0.01, compared with blank; n = 3.

Rescue experiments were performed to verify the function of hsa_circ_0091994 in GC cells. Since hsa_circ_0091994 knockdown inhibited GC cell proliferation via suppressing the miR-324-5p/HMGA1 axis, HMGA1 overexpression (OE) and shRNA-1 vectors were co-transfected into AGS cells. HMGA1 OE reversed the decrease in cell viability that was induced by shRNA-1 (Figure 5A). Similarly, HMGA1 OE reversed the increase in apoptosis that was induced by shRNA-1 (Figure 5B). Protein expression of HMGA1, Bax, Bcl-2, and cleaved caspase-3 was examined by western blot (Figure 5C). The shRNA-1 decreased HMGA1 and Bcl-2 expression, and increased Bax and cleaved caspase 3 levels (Figure 5D–5G). However, HMGA1 OE reversed all of these effects (Figure 5C–5G).

### Hsa_circ_0091994 knockdown inhibited tumor growth in mouse xenograft model

**Figure 6:**
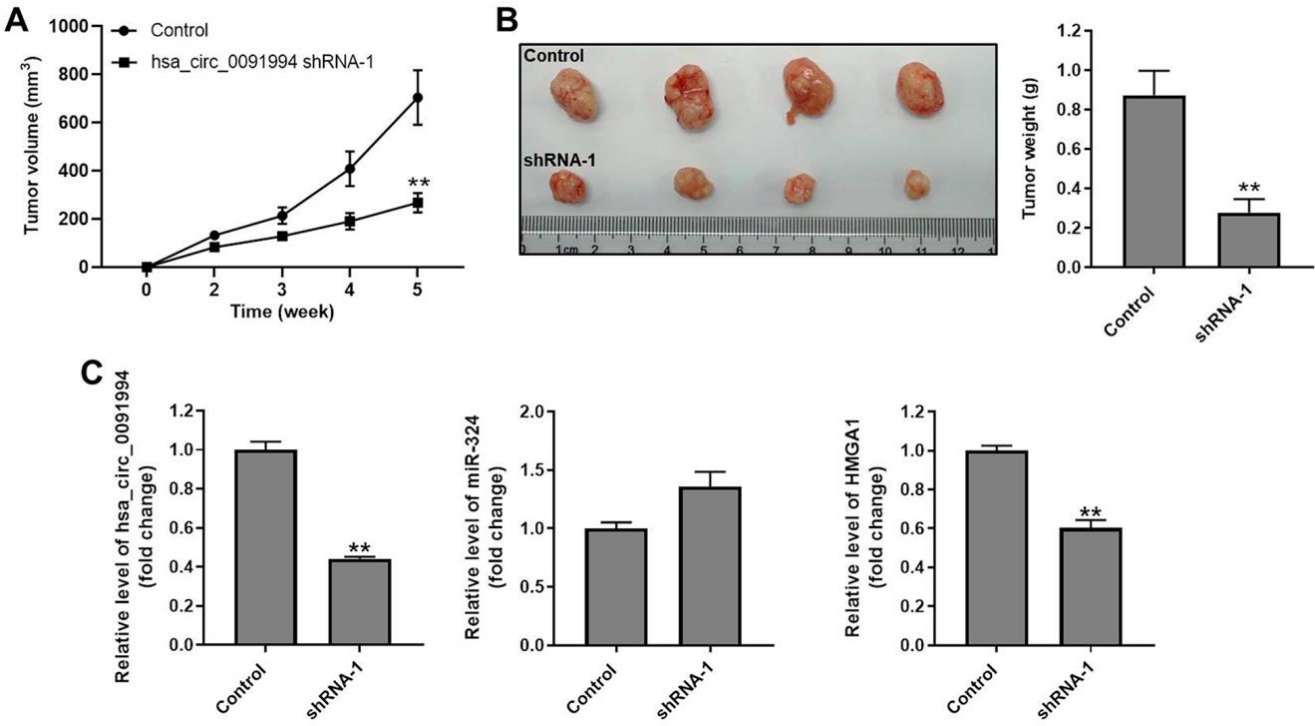
**Hsa_circ_0091994 knockdown repressed GC tumor growth in mouse xenograft model.** Hsa_circ_0091994 shRNA-1 treated AGS cells were injected into right dorsal of the mice (n = 6 per group). (**A**) Tumor volume was measured at weekly. (**B**) Tumor tissues were harvested and weighted at the end of week 5. (**C**) The expressions of hsa-circ-0091994, miR-324-5p and HMGA1 in tumor tissues were detected with RT-qPCR. **P<0.01, compared with control; n = 6.

Finally, the role of hsa_circ_0091994 was explored using with a mouse xenograft model. Tumor growth was suppressed by hsa_circ_0091994 knockdown at 5 weeks (Figure 6A). Tumor tissue was harvested and weighed right after sacrifice. Tumors in the shRNA-1 group were smaller than those in the control group (Figure 6B). In addition, the levels of hsa_circ_0091994 and HMGA1 in the shRNA-1 group were notably downregulated, while miR-324-5p level was slightly upregulated (Figure 6C). These data indicated hsa_circ_0091994 knockdown inhibited GC tumor growth *in vivo*.

## DISCUSSION

Gastric cancer (GC) patients typically have a poor prognosis [3, 4, 7, 9], and some circRNAs are biomarkers and therapeutic targets for cancer treatments [15–17]. We investigated hsa_circ_0091994 *in vitro* and *in vivo* because its parental gene FLNA expression was negatively associated with the overall survival of GC patients.

We found miR-324-5p was the downstream miRNA of hsa_circ_0091994 in GC, and increased miR-324-5p can inhibit GC progression [18]. Using StarBase database, we found HMGA1 to be the direct target of miR-324-5p, and HMGA1 has oncogenic activity in multiple cancers, including GC [21, 22]. Cell viability assay and a xenograft model showed that HMGA1 overexpression could reverse the anti-tumor effect of hsa_circ_0091994 knockdown in GC.

Hsa_circ_0091994 knockdown inhibited GC progression by suppressing the miR-324-5p/HMGA1 axis, which suggests hsa_circ_0091994 might serve as a novel therapeutic target for the treatment of GC.

## MATERIALS AND METHODS

### Bioinformatics analysis

The circRNAs expression data of GC tissues and matched normal tissues data were downloaded from the Gene Expression Omnibus (GEO, https://www.ncbi.nlm.nih.gov/geo/) under the accession number GSE141977. The differentially expressed circRNAs (DEcircRNAs) between GC tissues and normal tissues were analyzed and identified using R. P-value < 0.05 (-log_10_ p-value > 1.3) and |log_2_ Fold Change| > 1.5 was set as the threshold of significant DEcircRNAs. FLNA is the parental gene of hsa_circ_0091994. The association between the level of FLNA and the survival rate of patients with GC was analyzed using TCGA dataset (http://tcga-data.nci.nih.gov/tcga). GIEPIA (http://gepia.cancer-pku.cn/about.html) tool was employed to analyze the level of HMGA1 in GC tissue and normal gastric tissue.

### Cell culture

Human gastric epithelial cell line GES-1 was obtained from Beyotime (Shanghai, China). Gastric cancer cell line AGS was purchased from American Type Culture Collection (ATCC, Manassas, VA, USA). Gastric cancer cell line MKN45 and HGC-27 cells were obtained from Shanghai Institutes for Biological Sciences (SIBS, Shanghai, China). All cells were maintained in RPMI-1640 medium (Thermo Fisher Scientific, Waltham, MA, USA) supplemented with 10% FBS, 100 U/mL penicillin, and 100 mg/mL streptomycin in a humidified 5% CO_2_ incubator at 37° C.

### Sample collection

The gastric cancer tissues and adjacent normal tissues were isolated from 5 patients with gastric cancer in Henan Provincial People's Hospital. Tissues were isolated and stored in liquid nitrogen immediately. The study was approved by the Ethics Committee of Henan Provincial People's Hospital, and the written informed consent from patient was documented.

### Reverse transcription-quantitative PCR (RT-qPCR)

Total RNA Extraction Reagent (ELK Biotechnology, Wuhan, China) was used to extract total RNA from the cells and tissues. The related data of the tumor tissues were presented in Figure 6A–6C. Then, EntiLink™ 1st Strand cDNA Synthesis Kit (ELK Biotechnology) was employed for reverse transcription. The qPCR reactions were carried out with EnTurbo™ SYBR Green PCR SuperMix (ELK Biotechnology) on a StepOne™ Real-Time PCR System (Thermo Fisher Scientific). Thermal cycling protocol was pre-incubation at 95° C for 3 min, followed by 40 cycles of 95° C for 10 s, 58° C for 30 s, and 72° C for 30 s. Relative expression was determined using the comparative 2^-ΔΔCt^ method. U6 was used as the inner control for miRNA324-5p. β-actin acted as the inner control for other genes. Primers were listed as follows: β-actin, forward, 5′-GTCCACCGCAAATGCTTCTA-3′, reverse, 5′-TGCTGTCACCTTCACCGTTC-3′; Hsa-circ-0091994, forward, 5′-CGACCACCATGACAACACCTA-3′, reverse, 5′-CCAGCAGCTTTGGCATTTAC-3′; Hsa-miR-324-5p, forward, 5′-CGCATCCCCTAGGGCAT-3′, reverse, 5′-CTCAACTGGTGTCGTGGAGTC-3′; U6, forward, 5′-CTCGCTTCGGCAGCACAT-3′, reverse, 5′-AACGCTTCACGAATTTGCGT-3′.

### Establish cell lines with stable hsa_circ_0091994 knockdown

Short hairpin RNA (shRNA) against hsa_circ_0091994 and control shRNA (shRNA-ctrl) were synthesized and constructed into pcDNA3.1 vector by GenePharma (Shanghai, China). The pcDNA3.1 vector containing shRNA-ctrl, shRNA-1, or shRNA-2 was transfected into AGS and HGC-27 cells with Lipofectamine 2000 transfection reagent (Thermo Fisher Scientific). The transfection procedures were carried out per manufacturer’s instructions. Stably infected cells were selected using 5 μg/ml neomycin (Sigma, Saint Louis, MO, USA). The sequences of shRNA were as follows: shRNA-1, 5′-TGTCCAGCAGGTGTCGATTCAAGAGATCGACACCTGCTGGACATTTTT-3′; shRNA-2, 5′-GGTGTCGAGCTTGGCAATTCAAGAGATTGCCAAGCTCGACACCTTTTT-3′.

### Overexpression of HMGA1 and hsa_circ_0091994

The HMGA1 and hsa_circ_0091994 overexpression (HMGA1 OE and hsa_circ_0091994 OE) pcDNA3.1 vectors were constructed by Genepharma. AGS cells were transfected with pcDNA3.1-HMGA1-OE vector for 48 hr using Lipofectamine 2000 transfection reagent (Thermo Fisher Scientific). MKN45 cells were transfected with hsa_circ_0091994 OE for 48 hr as well.

### Cell viability assay

Cell counting kit-8 (CCK-8) assay was carried out to evaluate cell viability. Cells from each group were treated, then the cells were placed into a 96-well plate at density of 5×10^3^/ well. After 10 μl CCK-8 reagent (Beyotime) was added to each well, the cells were incubated with CCK-8 reagent at 37° C for 2 hr. The absorbance at 450 nm was determined using a spectrophotometer.

### Evaluation of cell apoptosis

The cells from each group were placed into 6-well plates at 3 × 10^5^ cell/well and cultured overnight. Annexin V-FITC Apoptosis Detection Kit purchased from Sungene Biotech (Tianjin, China) was employed for annexin V-FITC/propidium iodide (PI) double-staining, following the manufacturer’s protocol. Apoptotic cells were detected on a BD AriaIII flow cytometry system (BD Biosciences, Franklin Lake, NJ, USA).

### Scratch assay

Wound scratch assay was employed to examine the wound healing activity of AGS cells and HGC-27 cells after indicated treatment. The cells were placed into 6-well plate at 5 x 10^5^ cells/well. Once the cells created a confluent monolayer, scratches were created using sterilized 10 μL pipette tips. Then the cell monolayer was washed with PBS to remove cell debris. Wound gaps were photographed at 48 hr.

### Luciferase reporter assay

The potential downstream miRNA target of hsa_circ_0091994 was predicted using Starbase (http://starbase.sysu.edu.cn/) and CircInteractome (https://circinteractome.nia.nih.gov/index.html) online databases. The potential downstream target of miRNA was predicted with TargetScan (http://www.targetscan.org/vert_72/), miRWalk (http://zmf.umm.uni-heidelberg.de/apps/zmf/mirwalk/micrornapredictedtarget.html), and miRDB (http://www.mirdb.org/). Luciferase reporter assay was performed to verify the potential targeting relationship mentioned above. All oligos were synthesized by GenePharm (Shanghai, China). The oligos containing wild type or mutant 3’ UTR binding site of hsa_circ_0091994 were cloned into pMIR-reporter vector (Promega, Madison, WI, USA). Then, pMIR-reporter vector constructs, renilla luciferase reporter vector, and miR-324-5p agomir were co-transfected into AGS cells for 48 hr. Dual Luciferase Reporter Assay System (Promega, Madison) was used once the relationship between miR-324-5p and HMGA1 was verified. The results were presented as a ratio of firefly luciferase activity normalized to Renilla luciferase activity [23].

### RNA pull-down assay

RNA pull-down experiment was conducted according to previous reference [23]. Briefly, M-280 Streptavidin magnetic beads (Thermo Fisher Scientific) were incubated with the biotinylated miRNA-324-5p probe and negative control probe for 2 h at room temperature. After that, gastric cancer cells were harvested and incubated with probe-coated beads at 4° C overnight. Finally, the RNA complexes were extracted with Trizol reagent (Thermo Fisher Scientific) and analyzed by PCR assay.

### Western blot

The cells were lysed in RIPA buffer (Aspen Biotechnology, Wuhan, China) supplemented with Protease Inhibitor Cocktail (Roche, NJ, USA) for 5 min. The concentration of total protein was determined using BCA protein assay kit (Aspen Biotechnology). Total protein samples (40 μg) were separated by sodium dodecyl sulfate-polyacrylamide gel electrophoresis (SDS-PAGE). Separated proteins were blotted onto polyvinylidene fluoride (PVDF) membrane (Millipore, Bedford, MA, USA). After blocking in 5% skim milk at room temperature for 1 hr, the PVDF membrane was incubated with primary antibodies at 4° C overnight. The PVDF membrane was washed three times with tris-buffered saline and tween 20 (TBST) then incubated with HRP-Goat anti Rabbit secondary antibodies (1:10000, Aspen) for 2 hr at room temperature. The blots were visualized with an enhanced chemiluminescence (ECL) kit (Aspen). β-actin was utilized a loading control. The primary antibodies for western blot were: HMGA1 (1:1000, Abcam, Cambridge, MA, USA), Bax (1:2000, Cell Signaling Technology, Danvers, MA, USA), Bcl-2 (1:1000, Abcam), cleaved caspase-3 (1:1000, Abcam), β-actin (Abcam, 1:1000).

### Tumor xenograft model

Six week old BALB/c nude mice were obtained from Vital River (Beijing, China) and randomized into the control group and hsa_circ_0091994 shRNA-1 group (n=6 per group). AGS cells were transfected with hsa_circ_0091994 shRNA-1 for 48 h and then subcutaneously injected into the right flanks of the mice. Tumor volume was measured using a caliper every week after inoculation (Figure 6A). At the end of week 5, the mice were sacrificed, and tumor tissue was harvested (Figure 6B). The tumor weight was measured immediately after sacrifice (Figure 6B). The tumor volumes were calculated using 1/2 (length × width^2^). Meanwhile, the levels of hsa_circ_0091994, miR-324-5p and HMGA1 in tumor tissues of mice were detected by RT-qPCR (Figure 6C).

### Statistical analysis

The data were analyzed using GraphPad Prism 8.0 (GraphPad Software Inc., La Jolla, CA, USA). One-way analysis of variance (ANOVA) was utilized to examine the significance between groups. All data were presented as the mean ± SD. *P* < 0.05 was a statistical difference.

### Ethics approval and consent to participate

All animal procedures were approved by the ethics committee of Henan Provincial People's Hospital. The principles of the NIH Guide for the Care and Use of Laboratory Animals were strictly followed.

## Supplementary Material

Supplementary Figures
