# Supplementary material for: Knockdown of hsa_circ_0091994 constrains gastric cancer progression by suppressing the miR-324-5p/HMGA1 axis
Source: Aging (Albany NY). 2021 Aug 24;13(16):20598–608. doi: 10.18632/aging.203450 (PMC8436927; doi:10.18632/aging.203450)
Supplement: Supplementary Figures [file aging-13-203450-s001.pdf]

SUPPLEMENTARY FIGURES

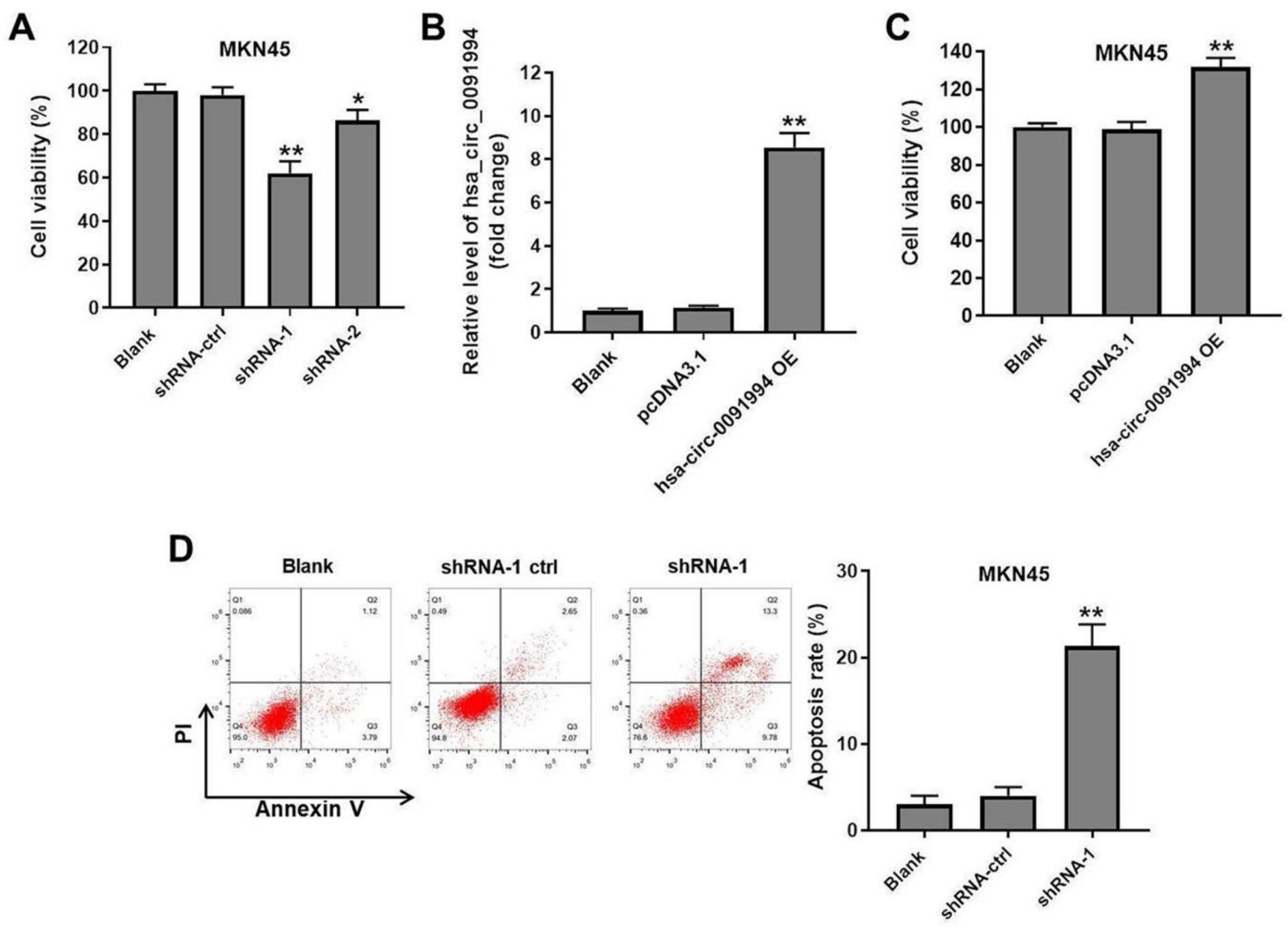

Supplementary Figure 1. Hsa_circ_0091994 knockdown inhibited the proliferation of MKN45 cells via inducing apoptosis. (**A**) MKN45 cells were administrated with shRNA-ctrl, shRNA-1 or shRNA-2 of hsa_circ_0091994 for 48 hr. The cell viability of MKN45 was determined by CCK-8 assay. MKN45 cells were administrated with hsa_circ_0091994 OE for 48 h. (**B**) The level of hsa_circ_0091994 was detected by RT-qPCR. (**C**) The cell viability of MKN45 was determined by CCK-8 assay as well. (**D**) MKN45 cells were administrated with shRNA-ctrl, shRNA-1 of hsa_circ_0091994 for 48 hr and cell apoptosis was measured in each group. *P<0.05, **P<0.01, compared with blank; n = 3.

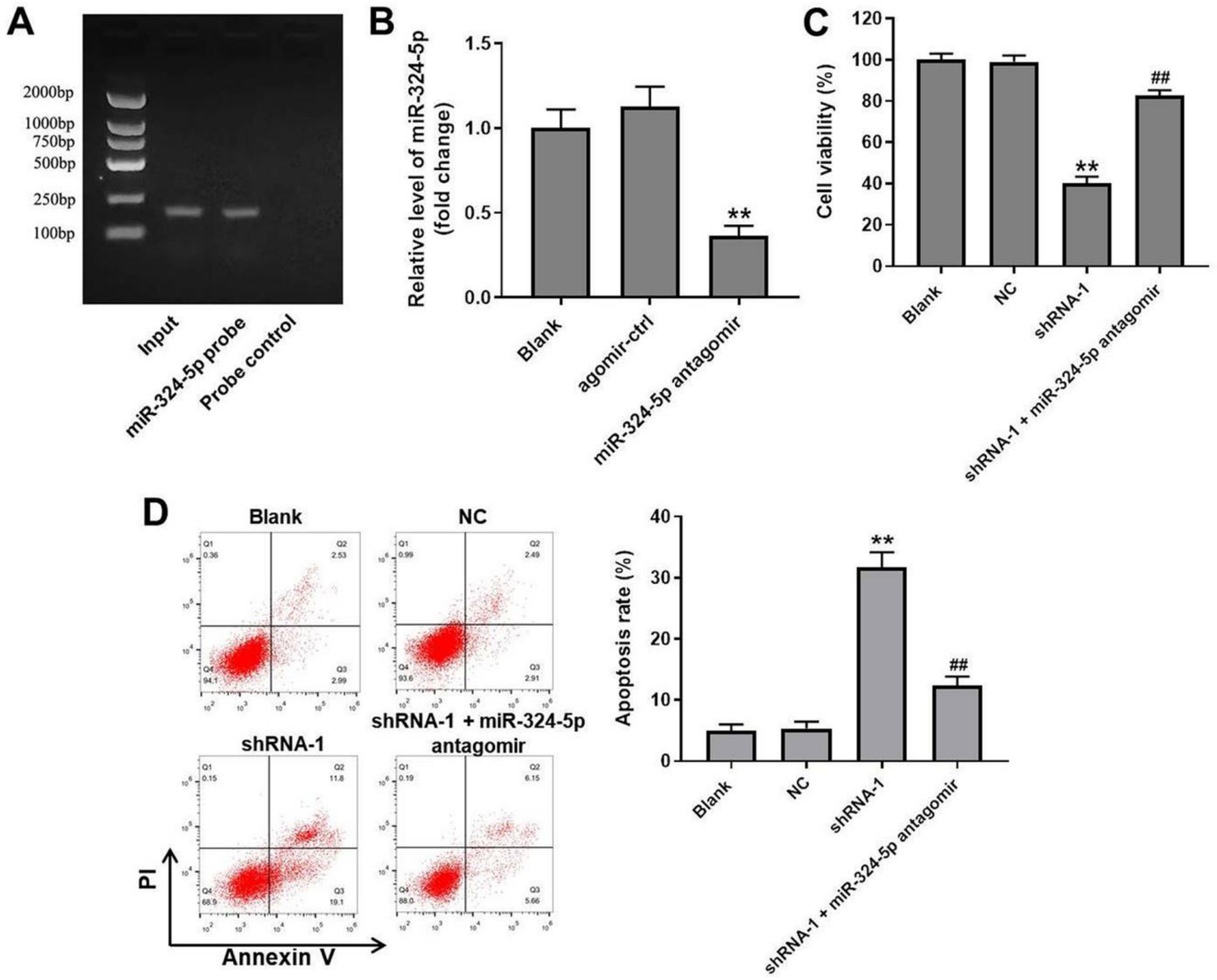

Supplementary Figure 2. The effects of hsa_circ_0091994 knockdown on the proliferation and apoptosis of MKN45 cells were reversed by miR-324-5p antagomir. (**A**) The interaction between hsa_circ_0091994 with miR-324-5p was detected with RNA pull down assay. (**B**) MKN45 cells were treated with miR-324-5p antagomir for 48 h; the level of miR-324-5p was measured with RT-qPCR. MKN45 cells were treated with hsa_circ_0091994 shRNA1 or/and miR-324-5p antagomir for 48 h. (**C**) Cell viability was measured with CCK8 assay. (**D**) Cell apoptosis was measured in each group. **P<0.01, compared with blank; n = 3.
